# Imaging Mild Cognitive Impairment and Dementia in Parkinson's Disease

**DOI:** 10.3389/fneur.2020.00047

**Published:** 2020-01-31

**Authors:** Sanskriti Sasikumar, Antonio P. Strafella

**Affiliations:** ^1^Division of Neurology, University of Toronto, Toronto, ON, Canada; ^2^Morton and Gloria Shulman Movement Disorder Unit & E. J. Safra Parkinson Disease Program, Neurology Division, Department of Medicine, Toronto Western Hospital, UHN, University of Toronto, Toronto, ON, Canada; ^3^Research Imaging Centre, Centre for Addiction and Mental Health, Campbell Family Mental Health Research Institute, University of Toronto, Toronto, ON, Canada; ^4^Division of Brain, Imaging and Behaviour – Systems Neuroscience, Krembil Research Institute, UHN, University of Toronto, Toronto, ON, Canada

**Keywords:** mild cognitive impairment, dementia, Parkinson's disease, biomarker, imaging

## Abstract

Cognitive dysfunction is a significant non-motor feature of Parkinson's disease, with the risk of dementia increasing with prolonged disease duration. Multiple cognitive domains are affected, and the pathophysiology cannot be explained by dopaminergic loss alone. Sophisticated neuroimaging techniques can detect the nature and extent of extra-nigral involvement by targeting neurotransmitters, abnormal protein aggregates and tissue metabolism. This review identifies the functional and anatomical imaging characteristics that predict cognitive impairment in PD, the limitations that challenge this process, and the avenues of potential research.

## Introduction

Parkinson's Disease (PD) is a neurodegenerative disorder that is often characterized by motor symptoms such as resting tremor, rigidity, bradykinesia, and postural instability. However, non-motor features such as cognitive impairment play a significant role in this progressive, multisystem condition, and often precede the onset of motor symptoms. In fact, mild cognitive impairment (MCI) is present in 42.5% of patients at the time of diagnosis, and progresses to dementia (PDD) in 70–80% of cases within 15–20 years (1, 2). The prevalence of dementia in PD is 3 times higher than the general population (3, 4), and leads to caregiver burden, early institutionalization and death (5).

The clinical syndrome in PD reflects the progressive degeneration of nigrostriatal dopamine neurons and accumulation of intranuclear misfolded alpha-synuclein, referred to as Lewy bodies. Executive dysfunction, that is a prominent feature of the cognitive dysfunction in PD, can be attributed to dopaminergic fronto-striatal impairment. However, other symptoms like alertness and visuo-perceptual dysfunction, cannot fully be explained by dopaminergic loss, and is suspected to involve several other neurotransmitter systems such as serotonergic, noradrenergic and cholinergic circuits. However, the role of extra-dopaminergic pathways in the cognitive symptoms in PD has not been fully elucidated and remains poorly understood.

The advent of sophisticated neuroimaging techniques has facilitated the detection of disease beyond the limitations of the clinical examination. Imaging biomarkers can be used as a potential tool to detect these extra-dopaminergic pathways, by specifically targeting neurotransmitters, abnormal protein aggregates and tissue metabolism (Table 1). They can also clarify the pathological burden that results in cognitive impairment, and can prognosticate the risk factors that determine the progression to dementia. This review highlights the imaging modalities that facilitate the detection of cognitive impairment and dementia in PD, and identify prognostic factors and molecular mechanisms that result in its pathology.

**Table 1:** Summary of imaging biomarkers in cognitive impairment and dementia in Parkinson's Disease, with their respective grades of evidence.

**Imaging modality**	**Functional and anatomical biomarkers**	**Summary of evidence**	**Level of evidence**
MRI	White matter hyperintensities	Compared to age-matched controls, white matter hyperintensities are not predictive of cognitive impairment in PD	Level 2
	Cortical thickness	Cortical thinning predicts the risk of dementia in PD-MCI	Level 2
	DTI	– In PD, there is increased mean diffusivity and decreased functional anisotropy in the hippocampus and bilateral frontal and temporal lobes –This further corresponds to impaired performance in verbal and visuospatial memory, semantic fluency and executive function	Level 3
	Resting state fMRI	– PD-MCI and PDD subjects demonstrate progressive loss of resting state nuclei in multiple brain regions – Visuospatial deficits in PD-MCI correlates with impaired functional connectivity in the parieto-temporal region, and is predictive of cognitive impairment	Level 2
	Task-specific fMRI	Impaired nigrostriatal and mesocortical dopaminergic pathways correspond to cognitive deficits in PD	Level 3
PET	11C-raclopride: dopamine D2-receptor marker 11C-dihydrotetrabenazine: D2-receptor and vesicular monoamine transporter (VMAT2) ligand marker	– Cognitive dysfunction in PD arises from nigrostriatal dopamine dysfunction and subsequent impairment of cortico-basal ganglia circuit – Reduction in D2 receptors in the insular cortex, anterior cingulate gyrus, and parahippocampal gyrus is seen in PD-MCI, with larger reduction seen in amnestic MCI subjects	Level 3
	Glucose (FDG-PET)	– PD cognition-related pattern (metabolic reduction in medial prefrontal, premotor and parietal association areas) identifies individuals with PD-MCI whose executive function will improve with levodopa –Individuals with PDD have more pronounced impairment of glucose metabolism in the primary visual cortex, with relative preservation of the medial temporal region	– Level 2 – Level 3
	^11^C-MP4A : acetylcholinesterase activity	– Cortical cholinergic activity is reduced by 30% in PDD and 11% in PD, and is associated with worse attention, memory and executive dysfunction	Level 3
	^11^C-PIB & 18F-FBB: bind to amyloid beta	– No difference in amyloid plaque burden between PD-MCI and healthy age-matched controls Predicts cognitive decline rather than reflecting cognitive impairment	Level 3
	[^18^F] AV-1451: binds to tau	– Conflicting reports about whether tau accumulation varies in PD-MCI compared to cognitive normal PD and age-matched controls –Presence of tau reflects more severe cognitive impairment, and usually accompanies the presence of amyloid beta	Level 3
	^11^C-PK11195: traces TSPO, expressed by activated microglia)	Inconsistent binding to midbrain, frontal and temporal cortices	Level 3
SPECT	^123^I-FP-CIT & [123I] ioflupane: *in vivo* marker of dopamine transporter binding	– Reduced uptake in striatum in DLB and PDD, and corresponds to low MMSE scores in PDD – Executive dysfunction could also be explained by impaired nigrostriatal function	Level 3
	^123^I-BVM: acetylcholine vesicle transport marker	Cholinergic activity is reduced in the parietal and occipital cortex in PD, with more extensive cortical reduction in PDD	Level 3
	(99m)Tc-ethyl cysteinate dimer (ECD): lipophilic agent used for SPECT perfusion imaging	– Global cortical hypoperfusion, most prominent in the temporo-parietal regions in PDD compared to healthy controls –Hypoperfusion in the posterior parieto-occipital regions in PD-MCI, with the most difference appreciated when compared to healthy controls and amnestic MCI than cognitively intact PD	Level 3

## Search Strategies and Selection Criteria

A search strategy was conducted using the PubMed database (www.ncbi.nlm.nih.gov/pubmed). The search criteria consisted of all English language articles published until September 23rd 2019, that contained combinations of “Parkinson,” “cognitive impairment,” “dementia,” “imaging,” “amyloid,” “tau,” “microglia,” “MRI,” and “PET,” The abstracts were screened for relevance, and read in their entirety if they were suitable.

## Magnetic Resonance Imaging (MRI)

### White Matter Hyperintensities

White matter hyperintensities (WMH) can be best visualized on the T2-weighted, fluid-attenuated inversion recovery (FLAIR) and proton density-weighted sequences, and their presence has been associated with worse cognition and postural stability in PD (6). However, previous studies have been inconsistent with the association of cognitive impairment and WMH.

White matter hyperintensities arise due to ischemic injury from either hemodynamic compromise, lipohyalinosis, or arteriosclerosis (7, 8). They are increasingly present with age, and are distributed in the deep subcortical white matter and periventricular regions (9, 10). Risk factors for their formation include hypertension, fluctuations in blood pressure and loss of cerebral vascular autoregulation (11). Because of their prevalence, it can be challenging to attribute pathology to their presence. A recent study by Pozorski et al. used an automated lesion segmentation tool to compare the WMH burden in PD to age matched controls over an 18-month period. Their findings reveal that PD patients do not experience more WMH than the average population, but the concurrent presence of WMH can exacerbate the cognitive and motor symptoms in the condition (12).

### Volumetric Assessment of Cortical Thickness

Atrophy in PD with mild cognitive impairment (PD-MCI) occurs predominantly over the frontal, temporal and parietal regions, as well as the basal forebrain, and it becomes more pronounced with progression to dementia (13). MRI analysis of cortical thickness can also be a means of prognosticating conversion to dementia. In a study that followed subjects with PD-MCI and healthy controls, cortical thinning in the frontal, left medial temporal and insular regions was associated with increased likelihood for MCI subjects converting to dementia (14). Loss of tissue volume in these regions is associated with impaired decision-making, executive function, recognition of facial expressions and visual memory (14). Voxel-based assessment of gray matter volume (GMV) corroborates this loss in the frontal-limbic and temporal regions, and suggests that unilateral-to-bilateral loss of GMV is a quantitative predictor for the progression from PD-MCI to PDD (15).

The cortex of PD-MCI converters is thinner than non-converters, despite the degree of cognitive impairment being comparable between the two groups (16). More cortical thinning poses an increased risk of developing dementia (16). Retrospective analysis of structural MRI imaging in cognitively intact individuals with PD demonstrated that cognitive impairment can be predicted based on the region of atrophy. PD-MCI occurred within 6 months of hippocampal atrophy (17), 18 months of temporal cortex atrophy (18), and 2 years of prefrontal cortex, insular and caudate atrophy (19, 20).

#### Diffusion Tensor Imaging (DTI)

DTI is an MRI-based imaging technique that measures the magnitude (mean diffusivity) and direction (fractional anisotropy) of water molecule flow along the white matter tracts. In PD, there is increased mean diffusivity and decreased fractional anisotropy predominantly in the bilateral frontal and temporal regions and hippocampus, suggesting damage to the white matter tracts in these regions (21–25). These manifest clinically with impaired performance in verbal and visuospatial memory, semantic fluency and executive function (22).

#### Resting State Functional MRI (fMRI)

Functional MRI measures the blood-oxygen-level dependent (BOLD) signal to reflect the functional connectivity (FC) in the brain. This provides information about the interaction between different cortical regions that make up resting-state networks (RSN). These fMRI images are obtained in the absence of prompted task, and demonstrate the resting connectivity in the brain.

PD subjects demonstrate a progressive loss of RSN in multiple brain regions over the course of a 3-year follow-up (26). Despite controlling for dopaminergic medications, subjects with PD-MCI and PDD demonstrate impaired FC in the fronto-parietal network and its associated RSNs (27, 28). In PD-MCI with visuo-spatial impairment, the FC changes involve the parieto-temporal regions as well, and is predictive of evolution to dementia (26, 29). These findings suggest a dual signature of cognitive impairment: the dopaminergic fronto-striatal pathway and the parieto-temporal pathway (30, 31).

### Task-Based fMRI

In individuals with PD, task-based MRI performed during attentional set-shifting tasks show reduced fronto-striatal activity (32–34). PD-MCI off dopaminergic medication further show diminished activation between the prefrontal cortex and caudate nucleus when performing set-shifting tasks (34). Some studies indicate that the medial temporal lobe compensates for this diminished functioning, and attempts to preserve cognitive function in PD (34–36). Task-based MRI studies demonstrate that the nigrostriatal and mesocortical dopaminergic pathways are involved in the cognitive deficits seen in PD (14).

## Positron Emission Tomography (PET)

### Dopamine

Dopaminergic molecular imaging has been useful in identifying the subcortical regions of dopamine hypoactivity that contributes to cognitive impairment in PD. By using a marker for dopamine D2-receptor availability and synaptic dopamine function (11C-raclopride, or RAC), Sawamoto et al. studied subjects with early symptomatic PD and age-matched controls during a spatial working memory task and visuomotor task to determine whether the cognitive impairment in PD results from frontal lobe or striatal dopaminergic dysfunction (37). They found that dopamine release in the dorsal caudate was significantly reduced in PD, but preserved in the medial prefrontal cortex, suggesting that cognitive deficits in early patients with PD arises from nigrostriatal dopamine dysfunction and subsequent impairment of the cortico-basal ganglia circuit (37).

Contrary to prior studies that implicated frontal dopamine depletion in cognitive impairment, there was relative preservation of mesocortical dopamine transmission (37). However, the studies that demonstrated diminished activity in the mesolimbic and mesocortical regions (38, 39), were done in PDD instead of early PD with MCI. So, it is possible that with disease progression, the cortical dopaminergic activity becomes more impaired.

By using a D2-receptor and vesicular monoamine transporter (VMAT2) ligands as a marker for dopamine receptor integrity in the cortex and striatum respectively, Christopher et al. demonstrated that cognitive impairment in PD is associated with D2 receptor reduction in the insular cortex, anterior cingulate gyrus and parahippocampal gyrus compared to cognitively intact PD controls. This reduction was also larger in amnestic compared to non-amnestic MCI subjects. This suggests that the medial temporal lobe contributes to the memory impairment and executive dysfunction in PD (40).

### Glucose

FDG-PET findings in PDD manifest in a similar pattern to Alzheimer's Disease, where the regional glucose cerebral metabolism affects the posterior cingulate gyrus first, then progresses to the temporal and parietal association cortex, and finally affects the prefrontal cortex (41, 42). Studies on PD-MCI also reveal diminished glucose metabolism in the parietal, temporal, cingulate, and frontal cortices (43, 44).

Despite similar findings in AD and PDD, the degree of regional cerebral hypometabolism can distinguish between the two conditions when matched for age, sex, and dementia severity. Individuals with PDD have pronounced impairment of glucose metabolism in the primary visual cortex, with relative preservation of the medial temporal region (45). However, in AD, there is only mild reduction in glucose metabolism in these regions (45).

Dementia with Lewy Bodies (DLB) is another neurodegenerative condition that shares similar PET imaging features with PDD. Compared to PD, PDD and DLB demonstrate relatively reduced metabolism in the bilateral inferior and medial frontal lobes, as well as the right parietal lobe (46). The only distinguishing feature is that glucose metabolism is more diminished in the anterior cingulate gyrus in DLB compared to PDD (46).

Spatial covariance analysis of FDG-PET images in PD reveals metabolic reductions in the medial prefrontal, premotor and parietal association areas in a pattern that is referred to as PD cognition-related pattern (PDCP). PDCP is associated with severe cognitive impairment, and can identify the population of patients with MCI whose executive function will improve with levodopa; those who do not demonstrate this pattern, do not respond to therapy (47).

### Acetylcholinesterase Activity

Cortical cholinergic deficiency plays a critical role in the cognitive decline seen in PD. Cholinergic denervation occurs early in PD, and is more pronounced in individuals with cognitive impairment and dementia (48–50). Lower cortical cholinergic activity is associated with worse cognitive performance scores for attention, memory, and executive function, and is sensitive enough to detect subclinical MCI (51). This justifies the use of cholinergic inhibitors for the symptomatic treatment of cognitive impairment in PDD (51).

While cortical cholinergic deficiency does not correlate with motor symptoms in PD, it appears to be the primary etiology associated with significant cognitive decline (48–50). Cortical binding of ^11^C-MP4A, a marker of acetylcholinesterase activity, is globally reduced by 30% in PDD, compared to 11% in PD (42). This pattern of cortical hypometabolism is also seen in DLB and AD, with AD demonstrating additional involvement of the thalamus (49, 51). Cholinergic PET also correlates with loss of forebrain cholinergic neurons seen on post-mortem pathology (48–50). ^18^F-dopa studies indicate that diminished dopaminergic uptake in the frontal and temporo-parietal regions correlates with cholinergic deficiency, suggesting that both systems reduce in conjunction in PD and PDD (48).

### Amyloid Beta

The role of amyloid beta in the development of cognitive impairment in PD is debated. Using the ^11^C-PIB marker, amyloid beta plaques can be identified in the cortex (52–54), but the degree is significantly less in PD compared to cognitively normal elderly people (55, 56). However, there is a higher plaque burden observed in the cortex and striatum of individuals with PDD (15–20%) and DLB (52, 57).

The presence of amyloid beta plaques is not presumed to be a major player in the pathogenesis of cognitive impairment in PD. There was no difference in amyloid beta burden between PD-MCI and healthy age-matched controls, but long-term follow up indicates that both groups experience cognitive decline at 2.5 years (58). This suggests that the presence of plaques might predict cognitive decline rather than reflect the severity of cognitive impairment (58). Additionally, the presence of amyloid plaque in the striatum is associated with worse cognitive decline compared to its presence in the cortex alone (59).

The *in vivo* amyloid beta PET imaging studies have several limitations; particularly, low sample size, low power, lack of cognitive characterization and no standardization for age (60). A recently published cross-sectional study by Melzer et al. (60) looked at 115 subjects divided into PD-CN, PD-MCI and PDD. Using 18F-Florbetaben (FBB) amyloid PET and structural MRI, the study determined that while FBB binding was higher in PDD population, it was not significant when adjusted to findings in age-matched controls, and is much lower than the deposition seen in AD (54, 61–63). This further suggests that amyloid beta is not the primary pathology that constitutes the cognitive impairment seen in PD.

### Tau

Tau deposits using tracer [^18^F] AV-1451 show increased binding in precuneus and inferior temporal gyrus in PDD and DLB, compared to age-matched controls (64, 65). However, in a recent cross-sectional study by Winer et al., tau accumulation did not differ between PD-CN and PD-MCI compared to age-matched controls (66). This suggests that cortical tau aggregates are present with more progressive disease only. While it is uncertain whether they contribute to cognitive dysfunction in PD, their presence certainly reflects more advanced cognitive impairment (51). Additionally, tau accumulation increases with amyloid beta, regardless of cognitive status and age (66).

### Microglia

Microglia are resident macrophages of the central nervous system that are activated during neuronal injury. They have been implicated in the pathogenesis of PD because post-mortem studies demonstrate increased microglia in the substantia nigra, putamen, hippocampus, cingulate and temporal cortex (67). As such, identifying microglial activity could serve as a biomarker of inflammation and potentially, PD.

Upon activation, microglia express TSPO (18 kDA translocator protein) in the outer mitochondrial membrane (68). However, PET studies using the ^11^C-PK11195 tracer show inconsistent binding to TSPO in the midbrain (69), frontal and temporal cortices (70). This variability might be explained by the limitations of the tracer itself, as ^11^C-PK11195 has non-specific binding, low blood-brain barrier penetration, and high plasma protein binding. It is also challenging to prepare, and these demonstrate different affinities because of polymorphisms in TPSO (71, 72).

Increased cortical microglial activation is associated with glucose hypometabolism in the posterior cingulate, frontal, temporal, parietal, and occipital regions in PD-MCI and PDD, suggesting that neuronal disconnections occur both locally and remotely (73). The degree of microglial activation also correlates with the cortical load of amyloid beta, so neuroinflammation may be an early event in dementia and likely initiates neuronal dysfunction (73).

## Single-Proton Emission Computed Tomography (SPECT)

### Dopamine

Similar to PET imaging, SPECT employs radiotracers to study the basal ganglia and its synaptic projections. ^123^I-FP-CIT SPECT, which is an *in vivo* marker of dopamine transporter (DAT) binding, shows reduced striatal uptake in individuals with DLB and PDD (74). In comparison, cognitively intact individuals with PD demonstrate more selective DAT binding to the putamen (75). Moreover, ^123^I-FP-CIT SPECT binding corresponds to MMSE scores in PDD patients, suggesting that striatal dopaminergic loss contributes to cognitive impairment in PD (74). SPECT studies using [123I] ioflupane suggest that executive dysfunction in PD could also be explained by impaired nigrostriatal function (76, 77).

### Acetylcholine

^123^I-BVM is a marker of acetylcholine vesicle transport, and reflects cholinergic deficiency in PD. Cognitively intact individuals with PD have reduced ^123^I-BVM binding in the parietal and occipital cortex, whereas those with PDD show extensive reduction in cortical binding (78).

### Perfusion

There is limited data on the role of SPECT perfusion imaging in PD-related cognitive impairment, but the reported literature suggests its potential in reflecting the severity of underlying cognitive impairment. A study of 22 patients with PDD using the SPECT radiotracer (99m)Tc-ethyl cysteinate dimer (ECD) reveals global cortical hypoperfusion, most prominent in the temporo-parietal regions, compared to healthy, age-matched controls (79). In individuals with PD-MCI, the hypoperfusion pattern is limited to the posterior parieto-occipital regions, with the most difference seen in comparison to healthy controls and amnestic MCI than to those with cognitively intact PD (80).

## Alpha Synuclein

The pathology of alpha-synuclein's invasion into the nervous system is unknown. It is theorized to originate in the enteric or peripheral nervous system, and enter the central nervous system through retrograde vagal nerve transport. However, not all PD subjects demonstrate pathology in the dorsal motor nucleus of the vagus to support this theory (81). Borghammer et al. hypothesize that there are two types of disease processes: (i) peripheral nervous system first, which manifests clinically with REM sleep behavioral disorder (RSBD) and (ii) central nervous system first, with nigrostriatal dopamine involvement prior to autonomic symptoms (81).

They support this theory using *in vivo* imaging of RSBD positive (idiopathic RSBD, who eventually develop PD or DLB) and RSBD negative (*de novo* H&Y Stage I PD patients) subjects. This revealed that RSBD positive subjects demonstrate severely reduced sympathetic cardiac innervation (using 123 I -MIBG scintigraphy) but normal striatal dopamine storage capacity (using 18 F-DOPA PET). RSBD negative subjects, who represent *de novo* PD, show normal cardiac innervation and reduced striatal dopamine capacity. These findings suggest that distinguishing RSBD reflects the initial location of the alpha synuclein pathology (81). Whether the disease starts in the enteric and peripheral nervous system vs. brainstem and limbic system might be determined by different patterns of cellular vulnerability (81). Unfortunately, there is no nucleotide tracer for alpha-synuclein, so much of its role in PD remains theoretical, as does its role in the progression of PD-MCI to PDD.

## Discussion

Emerging imaging techniques have facilitated a broader understanding of cognitive impairment, where extra-nigral pathways have been identified to play a key role in the pathogenesis of neurodegeneration in PD. Several mechanisms have been identified, including location of cortical atrophy as means of predicting risk of progression to dementia (17–20), meso-cortical dopaminergic dysfunction in advanced dementia, glucose hypometabolism in the posterior cingulate gyrus and temporo-parietal association cortices, and diffusely diminished cholinergic activity. Certain features like cortical deposition of amyloid beta and tau, as well as white matter burden, are associated with advanced cognitive impairment but do not participate in the primary pathology of cognitive impairment in PD. These processes act synergistically, and have poor prognostic implications for individuals with the disease (58, 82).

Cognitive impairment in PD starts in the temporo-parietal cortices before involving the remainder of the cortex and subcortical gray matter, and predicts the progression to PDD. There is diminished cholinergic activity in this region, as well as in the basal nucleus and occipital cortex, which justifies the use of cholinergic inhibitors for symptomatic treatment of attention and visuospatial disorientation in PD.

There are several limitations to the value of imaging to detect cognitive impairment in PD. Co-pathology of synucleinopathy (Lewy Body inclusions) and proteinopathy (amyloid beta and tau) is a feature shared with other dementing conditions like DLB and AD, which makes it challenging to distinguish PD-related cognitive impairment from these co-pathologies (83). It also remains unclear whether amyloid beta and tau influence the onset of cognitive impairment in PD, enhance existing symptoms or are predictive of its rate of progression. Studies are further limited by design and sample size, which challenges the ability to draw conclusions from their results. The average age of the patient population in the reported studies is over 55 years, with a disease duration between 8 and 10 years, so the relevance of these biomarkers to early-onset PD cannot be determined. There is also a need for new ligands with high affinity and better selectivity, that not only assess neurotransmission but also neuroinflammation (84). This will shed light on the series of events that take place in cognitive impairment, and determine the risk factors that drive this process. Identifying anatomical and functional predictors of cognitive impairment can facilitate early detection and target appropriate interventions to optimize patient care.

## Author Contributions

SS: execution and write-up of first draft of the manuscript. AS: conception of research subject and reviewing the manuscript.

### Conflict of Interest

The authors declare that the study was performed in the absence of commercial or financial relationships that could be construed as a potential conflict of interest.
